# Tailoring the Structure and Properties of Epitaxial Europium Tellurides on Si(100) through Substrate Temperature Control

**DOI:** 10.3390/ma16227093

**Published:** 2023-11-09

**Authors:** Fan Yu, Xiaodong Qiu, Jinming Zhou, Lin Huang, Bin Yang, Junming Liu, Di Wu, Gan Wang, Yi Zhang

**Affiliations:** 1 National Laboratory of Solid State Microstructure, School of Physics, Nanjing University, Nanjing 210093, China; dg1822056@smail.nju.edu.cn (F.Y.);; 2 Department of Physics, and Shenzhen Institute for Quantum Science and Engineering, Southern University of Science and Technology, Shenzhen 518055, China; 3 Collaborative Innovation Center of Advanced Microstructures, Nanjing University, Nanjing 210093, China; 4 Guangdong Provincial Key Laboratory of Quantum Science and Engineering, Shenzhen 518055, China; 5 Hefei National Laboratory, Hefei 230088, China

**Keywords:** EuTe, EuTe_4_, molecular beam epitaxy, thin film growth, structural characterization

## Abstract

In this study, we improved the growth procedure of EuTe and realized the epitaxial growth of EuTe_4_. Our research demonstrated a selective growth of both EuTe and EuTe_4_ on Si(100) substrates using the molecular beam epitaxy (MBE) technique and reveals that the substrate temperature plays a crucial role in determining the structural phase of the grown films: EuTe can be obtained at a substrate temperature of 220 °C while lowering down the temperature to 205 °C leads to the formation of EuTe_4_. A comparative analysis of the transmittance spectra of these two films manifested that EuTe is a semiconductor, whereas EuTe_4_ exhibits charge density wave (CDW) behavior at room temperature. The magnetic measurements displayed the antiferromagnetic nature in EuTe and EuTe_4_, with Néel temperatures of 10.5 and 7.1 K, respectively. Our findings highlight the potential for controllable growth of EuTe and EuTe_4_ thin films, providing a platform for further exploration of magnetism and CDW phenomena in rare earth tellurides.

## 1. Introduction

Rare earth tellurides (ReTe_x_) exhibit a diverse range of intriguing properties including charge density waves (CDW) [1,2,3,4], two-dimensional (2D) magnetism [5,6], as well as thermal hysteresis effects on resistivity and CDW gap [7,8], making them promising candidates for studying electron correlation phenomena and various applications in spintronics. Among these materials, europium tellurides are particularly unique due to the half-filled *4f* orbital of the Eu atom ([Xe] *4f^7^ 6s^2^*). According to Hund’s rule, the Eu atom possesses the maximum spin angular momentum with zero orbital angular momentum (*L* = 0, *J* = *S* = $\frac{7}{2}$). As a result, the magnetism in europium tellurides arises solely from the spin of the Eu atom.

**Figure 1:**
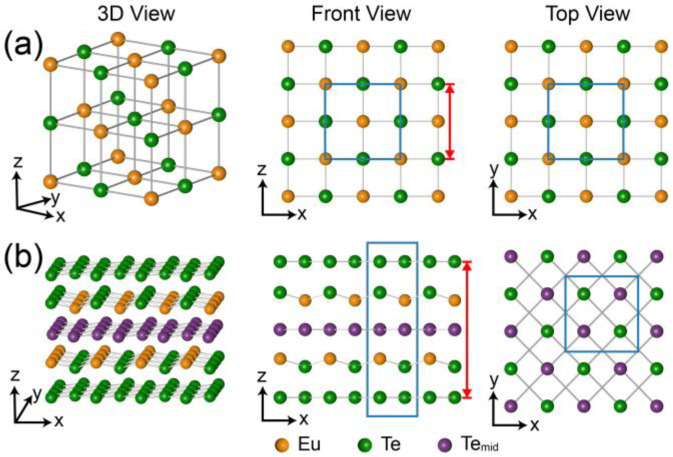
Three-dimensional (**left** panels), front (**middle** panels), and top views (**right** panels) of the (**a**) EuTe and (**b**) EuTe_4_ lattice. The yellow balls represent the Eu atoms, the purple balls represent the Te atoms in the middle layer (Te_mid_) of EuTe_4_, and the green balls represent the Te atoms in other positions. The blue solid rectangles indicate the respective unit cells, while the red double-headed arrows denote the single-layer thickness of both films.

Europium chalcogenides were among the earliest discovered magnetic semiconductors. In particular, EuTe, a renowned antiferromagnetic semiconductor, has been extensively studied for over half a century [9,10,11,12]. As shown in Figure 1a, it exhibits a face-centered cubic rock salt structure with a bulk lattice constant of *a* = *b* = *c* = 6.598 Å [13]. The valence state of Eu and Te in EuTe are +2 and −2, respectively [14]. In EuTe, the magnetic moments are carried by Eu, and the magnetic properties of EuTe can be depicted by an isotropic Hamiltonian, accounting only for the nearest and next-nearest neighbor exchange interactions [15,16,17]. At low temperatures, EuTe transitions into a type II antiferromagnet with a Néel temperature of 9.8 K [18].

In contrast, EuTe_4_ is a newly discovered van der Waals layered material that has received significant attraction in recent years [7,8,19,20,21]. As illustrated in Figure 1b, EuTe_4_ adopts an orthorhombic lattice structure with a space group of Pmmn (No. 59) at room temperature. The lattice constant of EuTe_4_ in its normal state are as follows: *a* = 4.5119(2) Å, *b* = 4.6347(2) Å, *c* = 15.6747(10) Å [20]. The unit cell of EuTe_4_ comprises a Te-EuTe-Te-EuTe-Te quintuple layer. The valence states of the Eu and Te ions in the Eu-Te slab are +2 and −2, respectively, while the valence state of the isolated Te layers remains nominally neutral [7]. The nearly square Te layers are unstable and tend to be distorted, resulting in CDW transition above 400 K [7]. Remarkably, EuTe_4_ exhibits a unique type of metastability, characterized by a thermal hysteresis that spans over 400 K in temperature. More specifically, the CDW gap and electrical resistivity of EuTe_4_ manifest different behaviors even at an identical temperature, depending upon the preceding temperature variation path (for instance, whether it was heated up to 300 K or cooled down to 300 K) [7]. The origin of this thermal hysteresis deviates from conventional mechanisms and can be elucidated by the switching of CDW phases in distinct Te layers, a phenomenon not present in 2D or strongly correlated 3D systems [7].

Despite extensive research on EuTe, the high-quality synthesis of EuTe thin films remains a challenge. Previous studies used BaF_2_(111) as the growth substrate, but the large lattice mismatch between EuTe film and BaF_2_ substrate necessitated the incorporation of PbTe(111) film as a buffer layer [22,23,24,25,26]. Moreover, the film quality was highly sensitive to the substrate temperature and required a rigorous flux ratio control. Furthermore, the thickness of EuTe film grown on BaF_2_ was also limited to 45 layers due to the formation of a strain-induced three-dimensional island [22]. Therefore, it is necessary to improve the growth procedure of EuTe in order to attain a deeper comprehension of the rich magnetic properties within this system. On the other hand, the current research on EuTe_4_ primarily focuses on its bulk properties, lacking a systematical investigation on EuTe_4_ thin film in a 2D limit. The synthesis of 2D epitaxial EuTe_4_ thin film serves as a platform to facilitate our understanding of the mechanisms behind its CDW behavior in subsequent studies. Also, it offers an opportunity to delve into the competitive interactions between different Te atomic layers, unraveling the underlying mechanisms that drive the thermal hysteresis phenomena.

Substrate temperature is a key factor in the molecular beam epitaxial (MBE) growth of thin films. Typically, substrate temperature only affects the morphology and quality of the films [27,28,29]. In specific instances, such as MoS_2_ [30], WSe_2_ [31], and TaTe_2_ [32], precise temperature control enables the selective growth of films with different crystalline structures. By carefully tuning the substrate temperature, one can manipulate the microstructure of the material at the atomic level. Such control paves new paths to precisely tailor the optical, magnetic, and electronic properties of the films.

In this research, we improved the growth procedure for EuTe and realized the epitaxial growth of EuTe_4_ utilizing MBE. The selective growth of EuTe and EuTe_4_ was also achieved by precisely adjusting the substrate temperature. In combination with reflection high energy electron diffraction (RHEED), X-ray diffraction (XRD), and scanning transmittance electron microscopy (STEM) techniques, we examined the difference of lattice structures and crystalline orientations between EuTe and EuTe_4_ films. In addition, we compared the relative stoichiometry ratio and valence state between EuTe and EuTe_4_ films via X-ray photoelectron spectroscopy (XPS). The experimental data demonstrated that a substrate temperature of 220 °C results in the growth of EuTe, and conversely, EuTe_4_ film forms at a lower temperature of 205 °C. Additionally, we further investigated the physical properties of the two materials. The XPS spectra near the Fermi level indicated that EuTe is a semiconductor, with its valence band top located about 0.6 eV below the Fermi level. For EuTe_4_, the density of states stretches to the vicinity of the Fermi level. The transmittance spectra verified the semiconductive property of EuTe and discovered the existence of a CDW gap in EuTe_4_ at room temperature. In addition, superconducting quantum interference device (SQUID) measurements denoted that EuTe and EuTe_4_ are both antiferromagnetic materials, with Néel temperatures of 10.5 and 7.1 K, respectively. Our results developed the fabrication and physical property investigation of epitaxial 2D materials based on rare earth elements.

## 2. Methods

The growth of EuTe and EuTe_4_ films was conducted in an MBE system (GC inno, Changzhou, Jiangsu, China) with a base pressure of 1 × 10^−10^ mbar. The conductive Si(100) wafers (*n*-type boron doped, 0.01~0.05 Ω·cm, HF-Kejing, Hefei, Anhui, China) were selected as substrates. Prior to the growth, the substrates underwent a degassing process at 600 °C for 3 h, followed by a standard flash procedure at 1200 °C to achieve an atomic flat surface [33]. The films were grown by co-deposited high-purity Eu (99.9%) and Te (99.999%) shots (PrMat, Shanghai, China) via standard Knudsen Cells on the Si(100) substrate. The temperatures of the evaporation sources for Eu and Te were maintained at 460 °C and 320 °C, respectively, with flux ratio of Eu:Te keeping ~1:20. The growth of the film was monitored by an in situ RHEED and the growth rate of EuTe and EuTe_4_ was about 0.3 and 0.2 nm per minute (nm/min), respectively. The thickness of the grown film, defined as the length in the z-direction of Figure 1a,b, was roughly estimated by the growth time.

The crystal structure of the grown films was determined by an ex situ XRD (D8 ADVANCE, Bruker, Billerica, MA, USA) with Cu Kα source (wavelength λ = 1.5406 Å). A spherical aberration-corrected scanning transmission electron microscopy (STEM, Titan Themis G2, FEI, Hillsboro, OR, USA) was employed for further examining the structure of the grown films. To protect the films from possible oxidation in atmosphere and ensure the sample was grounded during the STEM measurements, a ~20 nm thick amorphous Eu metal film was deposited on the sample surface at room temperature before moving the sample from the MBE chamber. The samples were fabricated by the focused ion beam (FIB, Helios Nanolab 600i, FEI, Hillsboro, OR, USA) technique before STEM characterizations. The stoichiometric information of EuTe and EuTe_4_ were compared by an in situ XPS with a resolution of ~0.2 eV, where the monochromatic X-ray (Al Kα, 1486.7 eV) was used as the excitation light source (Scienta Omicron MECS, Taunusstein, Hesse, Germany). The ex situ transmittance spectra of EuTe and EuTe_4_ were measured at room temperature, with light incident perpendicular to the sample surface and data collected by a Fourier transform spectrometer (Vertex 80 V, Bruker, Billerica, MA, USA). The magnetic properties were characterized by an ex situ superconducting quantum interference device vibrating sample magnetometer (SQUID-VSM, Quantum Design, San Diego, CA, USA). During the magnetic measurement, a magnetic field of 5000 Oe was applied parallel to the film surface.

## 3. Results and Discussions

### 3.1. Growth and Structural Characteristics of EuTe and EuTe_4_ Films

**Figure 2:**
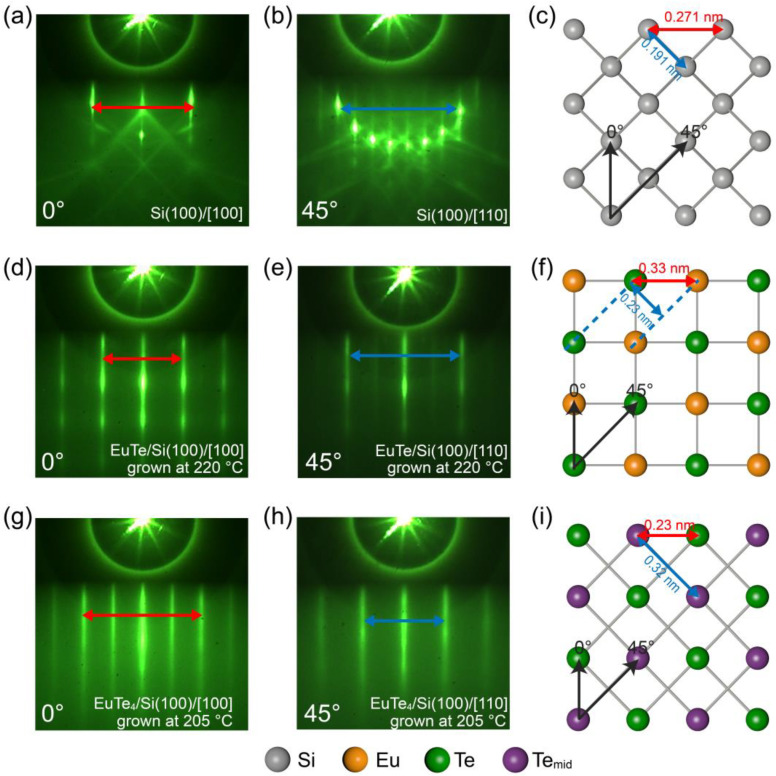
(**a**,**b**) RHEED patterns of a Si(100) substrate with incident beam angles of 0° and 45°, respectively. (**c**) Corresponding top view lattice arrangement for Si(100). The black arrows in (**c**) represent the incident direction of electron beams, while the blue and red arrows between atoms denote the RHEED diffraction stripe spacings as indicated in (**a**,**b**). The in-plane lattice constants, derived from the subsequent STEM analyses, are also annotated in the lattice arrangement diagram of (**c**). (**d**–**i**) Analogous to (**a**–**c**) for (**d**–**f**) a ~10 nm EuTe film and (**g**–**i**) a ~10 nm EuTe_4_ film.

Figure 2a displays the RHEED pattern of a 2 × 1 surface-reconstructed Si(100) substrate after the standard flash procedure, with the electron beam incident along the Si<100> direction. To clarify the lattice orientations of the substrate and grown films, we present the 45°-rotated RHEED diffraction pattern of Figure 2a in Figure 2b, where the electron beam incident is along the Si<110> direction. Figure 2c provides a schematic diagram of the atomic arrangement on the Si(100) surface. The black arrows indicate the incident directions (0° and 45°) of the RHEED electron beam, while the red and blue double-headed arrows correspond to the space between the diffraction stripes indicated in Figure 2a,b, respectively.

The substrate temperature played an essential role in determining the structural phase of the grown film. Figure 2d,e present the RHEED diffraction patterns of a ~10 nm thick film grown at 220 °C with the incident electron beam along the Si<100> (0°) and Si<110> (45°) directions, respectively. This film is further identified as EuTe by the subsequent XRD and STEM characterizations. The symmetry of the EuTe RHEED patterns matches that of the silicon substrate, demonstrating a four-fold rotational invariance. This rotational symmetry indicates the grown film possesses a tetragonal structure with the EuTe(001) as the surface orientation, which is notably different from the scenario of EuTe grown on a BaF_2_(111) substrate with PbTe as a buffer layer, where the surface orientation of the EuTe/PbTe/BaF_2_ film is the (111) plane, displaying a six-fold rotational symmetry [13]. Moreover, as the thickness of the film increases, no evidence of 3D island growth was observed, contrasting with the behavior of the EuTe film on BaF_2_(111) substrates, where 3D roughness rapidly increases when reaching the critical layer thickness of 45 layers [24]. This result suggests greater stability and lower binding energy for the EuTe(001) plane [34].

In Figure 2f, the atomic arrangement of the EuTe(001) surface is illustrated, with Eu and Te atoms represented by yellow and green balls, respectively. The RHEED diffraction stripes in Figure 2d (indicated by the red double arrow) correspond to the spacing between adjacent Eu and Te atoms as indicated by the red double arrow in Figure 2f. Similarly, the diffraction stripes in Figure 2e (indicated by the blue double arrow) correspond to the spacing between adjacent Eu(110) and Te(110) planes of EuTe in Figure 2f.

Lowering the substrate temperature to 205 °C results in the growth of EuTe_4_. Figure 2g,h display the RHEED pattern of a ~10 nm thick EuTe_4_ film along the Si<100> and <110> directions, respectively. The RHEED diffraction patterns of EuTe_4_ exhibit a four-fold rotational symmetry, indicating that the thin film’s surface orientation is the (001) plane, which is consistent with the cleavage plane of bulk EuTe_4_ observed in previous angle-resolved photoemission spectroscopic (ARPES) experiments [7,19]. In Figure 2i, a top view of the EuTe_4_(001) surface is presented, with the green and purple balls representing the topmost and middle Te atomic layers indicated in Figure 1b, respectively. The red and blue double-headed arrows in Figure 2g,h correspond to half of the basis vector along the *x*-axis and the nearest Te atom spacing within a single Te layer, as indicated in Figure 2i, respectively.

A substrate temperature significantly higher or lower than the optimal growth conditions for EuTe and EuTe_4_ will result in the degeneration of the film quality. More detailed results of the films grown at various temperatures can be seen in Supplementary Material Part A.

**Figure 3:**
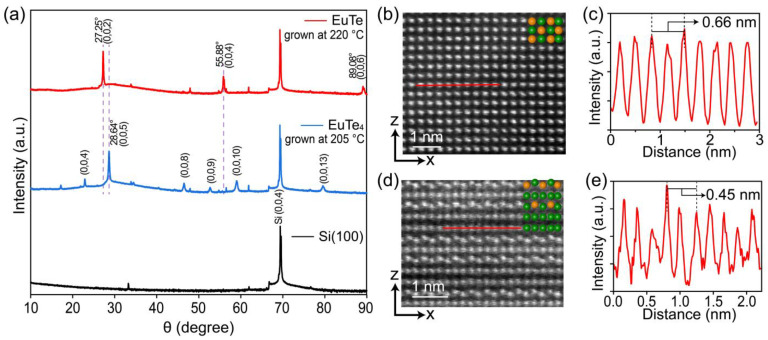
(**a**) XRD patterns of a Si(100) substrate (black curve), a ~20 nm EuTe_4_ film (blue curve), and a ~20 nm EuTe film (red curve). The vertical purple dashed lines serve as guides to highlight the spectral differences between EuTe and EuTe_4_. (**b**) STEM image (side-view) for a ~10 nm EuTe film, corresponding to the (010) plane of EuTe. (**c**) Intensity profile corresponding to the red solid line in (**b**). (**d**,**e**) Analogous to (**b**,**c**) for a ~10 nm EuTe_4_ film.

We carried out ex situ XRD and STEM characterizations to further identify the crystal structure of the grown films. Figure 3a presents the XRD curves for the Si substrate (black curve), and the films grown at 205 °C (blue curve) and 220 °C (red curve), shown from bottom to top. To display the diffraction peaks with varying intensities on a unified scale, we applied a fourth-root adjustment to the XRD curve intensities. The principal diffraction peaks in each of the three spectra are annotated with their corresponding diffraction indices.

Aside from the dominant peak at 69.40°, which is attributed to the silicon substrate, the XRD diffraction curves of the films grown at 205 °C and 220 °C display notable differences. For the film grown at 220 °C, the peaks at 27.25°, 55.88°, and 89.08° correspond to the EuTe(002), (004), and (006) planes, respectively. This diffraction pattern is distinct from the XRD curve of EuTe(111) grown on the PbTe buffer layer on the BaF_2_(111) substrate, where the (222) peak is predominant [13,35]. From the XRD curve, we derived a lattice constant of *c* = 0.654 nm for EuTe by applying Bragg’s law. This value is very close to the lattice constant of 0.650 nm derived from the EuTe film grown on the PbTe layer on the BaF_2_(111) substrate [13], confirming that they are the same material but with different crystal orientations.

In contrast to the diffraction pattern of EuTe, EuTe_4_ exhibits the strongest peak at 28.64°, which is associated with the EuTe_4_ (005) plane and gives a lattice constant of *c* = 1.557 nm. This value is consistent with the lattice constant of 1.567 nm obtained from the XRD measurement of the bulk EuTe_4_ sample [20]. Based on the above XRD curves, we can conclude that the films grown at ~205 °C and ~220 °C belong to different structural phases of EuTe_4_ and EuTe, respectively.

Figure 3b displays the side-view STEM image of the EuTe film (grown at ~220 °C), where atoms form a tetragonal lattice. A schematic atomic arrangement of EuTe is depicted in the top-right corner of the STEM image. The intensity distribution curve, shown in Figure 3c, derived along the red solid line in Figure 3b, reveals an in-plane lattice constant of *a* = 0.66 nm for EuTe. This is in line with the value of 0.65 nm for EuTe grown on the PbTe buffer layer on BaF_2_(111) [13]. According to the lattice configurations depicted in Figure 2c,f and the derived in-plane lattice constant, we obtained a lattice mismatch of 21.55% between EuTe and the silicon substrate, which significantly surpasses the value of 2.10% between EuTe(111) and the buffer layer of PbTe on BaF_2_(111) [13]. The large lattice mismatch indicates a weak interfacial interaction between the substrate and the thin film, ensuring the high-quality growth of EuTe.

Figure 3d is a side-view STEM image of the EuTe_4_ film (grown at ~205 °C), which displays a layered atomic structure comprised of EuTe-Te-EuTe-Te-Te. The corresponding intensity distribution curve in Figure 3e yields an in-plane lattice constant of *a* = 0.45 nm for EuTe_4_, consistent with the value of 0.451 nm derived from bulk EuTe_4_ XRD characterization [20]. This result gives a lattice mismatch of 17.13% between the Si substrate and EuTe_4_ film.

### 3.2. XPS Differences in EuTe and EuTe_4_

**Figure 4:**
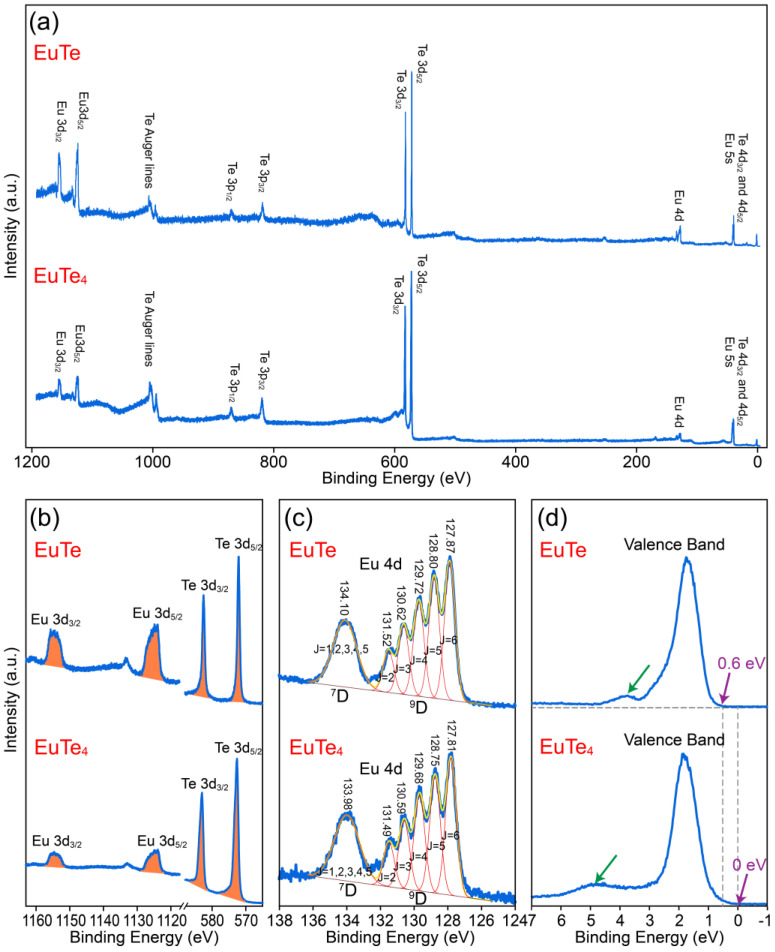
XPS spectra for ~10 nm EuTe (**top** of each panel) and EuTe_4_ (**bottom** of each panel) films, including (**a**) the full range spectra, (**b**) the spectra for Eu *3d* and Te *3d* orbitals, (**c**) the spectra for Eu 4d orbital, and (**d**) the spectra near Fermi level. The horizontal gray dashed line in (**d**) denotes the baseline of EuTe spectra, while the vertical gray dashed lines indicate the cutoff energy of EuTe and EuTe_4_, respectively.

The elemental stoichiometry and valence states of EuTe and EuTe_4_ films were investigated by in situ XPS, with all measurements performed at 300 K. Figure 4a illustrates the full-range XPS spectra for EuTe (upper section) and EuTe_4_ (lower section). The two spectra exhibit significant differences, with the signal intensity of Eu *3d_3/2_* and Eu *3d_5/2_* orbitals in EuTe being notably higher than that in EuTe_4_, indicating a higher concentration of Eu in EuTe. To study the ratio of Te to Eu in EuTe and EuTe_4_, we present a detailed scan of Eu *3d_3/2_*, Eu *3d_5/2_*, Te *3d_3/2_*, and Te *3d_5/2_* orbitals in Figure 4b. The relative peak areas of each peak after background subtraction are listed in the left half of Table 1, where the peak area of the Te *3d_5/2_* orbital was normalized as unit one. It shows that the peak area ratios of the *3d_3/2_* to *3d_5/2_* orbitals for each element in both the EuTe and EuTe_4_ closely match a 2:3 distribution, which is in line with the characteristics of *p*-orbital electrons in XPS spectra, indicating our treatment of peak areas is accurate. The right half of Table 1 lists the comparative area ratios of Eu’s individual *3d* orbital to those of Te *3d* orbitals. We further calculated the quotient of this ratio in EuTe relative to that in EuTe_4_, as shown in Table 2. This result provides a representation of the relative Te content in EuTe_4_ compared to EuTe. The resultant value ranges from approximately 3.79 to 3.93, closely approximating 4, which implies the Te content in the EuTe_4_ is about four times compared to that in EuTe. The error primarily stems from the different photon–electron cross-sections of the different Te elements in EuTe and EuTe_4_.

Figure 4c displays the detailed scanning spectra along with the fitting curves of Eu *4d* orbitals in EuTe and EuTe_4_, respectively, where the position of each peak is listed above the corresponding curve The leftmost peak of each Eu *4d* orbital comprises five orbitals *^7^D_1_*, *^7^D_2_*, *^7^D_3_*, *^7^D_4_*, and *^7^D_5_*, and the five peaks on the right represent the five orbitals *^9^D_2_*, *^9^D_3_*, *^9^D_4_*, *^9^D_5_*, and *^9^D_6_* [36]. The *4d* orbital spectra in EuTe and EuTe_4_ show similar peak shapes and positions within the experimental error range, further confirming that the valence state of Eu remains unchanged (+2 state) in EuTe and EuTe_4_.

Meanwhile, we present the XPS spectra near the Fermi level in Figure 4d. The peak width and position of their respective leftmost peaks (highlighted by the green arrows) exhibit distinct differences. Specifically, this peak in EuTe_4_ exhibits a broader width compared to that in EuTe, and its peak position shifts to a deeper binding energy. Moreover, the spectrum of EuTe cuts off at ~0.6 eV, indicating that EuTe is a semiconductor with its valence band maximum located ~0.6 eV below the Fermi level. This is consistent with the 2.26 eV band gap observed in EuTe on the BaF_2_(111) substrate [37]. In contrast, the spectrum for EuTe_4_ stretches close to the Fermi level. From an energy band theory perspective, we can infer from the XPS spectrum that EuTe_4_ exhibits a density of states in the vicinity of the Fermi level (from –0.2 to 0 eV). Considering that our XPS resolution is ~0.2 eV, this result suggests that EuTe_4_ is either a small gap semiconductor or a metallic material. Previous ARPES results reported that EuTe_4_ has a CDW gap of ~0.2 eV at the Fermi level [7,8,19]. This value aligns well with our XPS measurements, especially considering the XPS resolution of ~0.2 eV.

### 3.3. Physical Property Characterizations of EuTe and EuTe_4_

**Figure 5:**
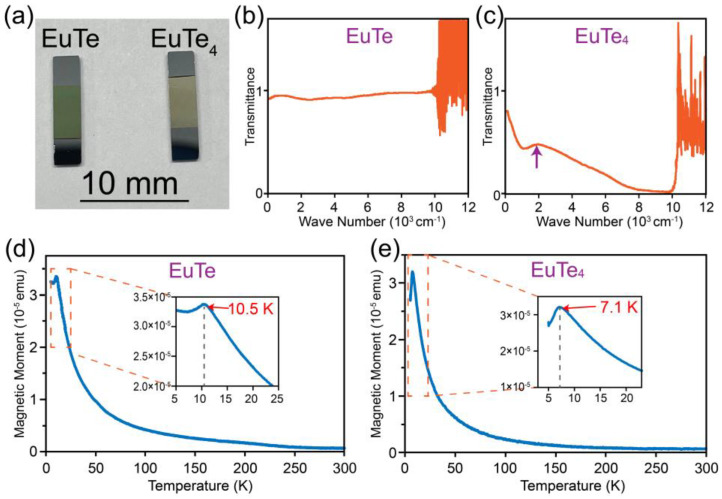
(**a**) Photograph of the epitaxial EuTe and EuTe_4_ films. (**b**,**c**) Transmittance curves of ~20 nm thick EuTe and EuTe_4_ film, respectively. (**d**,**e**) M−T curves of ~40 nm thick EuTe and EuTe_4_ film, respectively. Insets in (**d**,**e**) are the corresponding magnified curves at low temperatures.

We conducted ex situ transmittance spectroscopy on the two films, which is a widely used technique in probing the band gap of a material [37]. Figure 5a displays a photograph of EuTe and EuTe_4_ films before transmittance spectroscopy characterization. Notably, the films exhibit distinctly different colors, where EuTe appears green and EuTe_4_ is golden yellow, indicating the transmittance spectra of the two films are different.

Figure 5b,c present the transmittance spectra of EuTe and EuTe_4_, respectively. The process of transmittance spectroscopy characterization involves two steps. We first measured the transmittance spectrum of the apparatus and silicon substrate, represented as T_1_ = T_appa_ × T_sub_, which serves as a reference value. The result of T_1_ is shown in Supplementary Material Part B. Then we measured the transmittance spectrum of the apparatus, silicon substrate, and film: T_2_ = T_appa_ × T_sub_ × T_film_. Dividing the two values yields the transmittance spectrum of the film: T_film_. The band gap of our silicon substrate is approximately 1.1 eV, which corresponds to a wavenumber close to 10,000 cm^−1^. As a result, the transmittance spectrum of silicon substrate drops sharply to zero beyond this value. This leads to an indeterminate form in the T_film_ expression, resembling 0/0. Consequently, the transmittance spectrum for our film diverges above ~10,000 cm^−1^, making the data valid only for a wavenumber below this threshold.

In Figure 5b, the transmittance spectrum for EuTe is flat and remains close to 1 within the experimentally accessible range, indicating that EuTe is a semiconductor with a band gap exceeding 1.1 eV. This is consistent with the earlier optical transmittance measurement on EuTe grown on the BaF_2_ substrate, which identified a band gap of 2.26 eV [37].

In contrast, the transmittance curve for EuTe_4_ in Figure 5c presents a distinct absorption edge around 1900 cm^−1^ (highlighted by the purple arrow), corresponding to an energy of approximately 0.23 eV. Previous ARPES experiments have demonstrated the presence of a gap in EuTe_4_ induced by CDW at room temperature, with a size of ~0.2 eV [7,8,19]. This matches the energy of the absorption edge observed in our transmittance spectrum. Thus, this absorption edge is a manifestation of the CDW gap, specifically attributed to optical electron excitations across the CDW gap of ~0.23 eV.

Figure 5d,e are the magnetic moment versus temperature (M−T) curves of EuTe and EuTe_4_. The shapes of the two curves are very similar, both exhibiting sharp peaks at low temperatures, which is a typical feature of antiferromagnetic material. Here, we magnified and plotted the details of these curves at low temperatures in the inset. Consequently, we can derive the Néel temperatures of EuTe and EuTe_4_ to be 10.5 and 7.1 K, respectively, which are consistent with the 9.8 and 7.1 K reported in previous magnetic susceptibility measurements [18,20].

## 4. Conclusions

In conclusion, we have successfully synthesized high-quality EuTe and EuTe_4_ thin films on Si(100) substrates. Our study improves the growth procedure of epitaxial EuTe films and fills the research gap in the synthesis of two-dimensional EuTe_4_ films. We conducted a comprehensive study on the structural and energy spectra characterization of the two materials, confirming their antiferromagnetic nature. We also verified the semiconductive property of EuTe and found the CDW signature of EuTe_4_ at room temperature. By tailoring the substrate temperature, we have achieved selective growth of these two materials, opening new possibilities for their physical property control. Our research on rare earth tellurides has enriched the library of 2D materials. The high-quality growth of the film paves the way for subsequent related research such as 2D magnetism and charge density waves, and also facilitates the exploration of potential applications in electronics.

## Figures and Tables

**Table 1:** Normalized peak areas of Eu and Te *3d* orbits and their comparative ratios.

	EuTe	EuTe_4_		EuTe	EuTe_4_
Eu *3d_3/2_*	0.934	0.238	Te *3d_3/2_*:Eu *3d_3/2_*	0.718	2.719
Eu *3d_5/2_*	1.423	0.362	Te *3d_5/2_*:Eu *3d_3/2_*	1.071	4.211
Te *3d_3/2_*	0.671	0.646	Te *3d_3/2_*:Eu *3d_5/2_*	0.471	1.782
Te *3d_5/2_*	1	1	Te *3d_5/2_*:Eu *3d_5/2_*	0.703	2.760

**Table 2:** Ratio of Te content in EuTe_4_ to EuTe, based on the data in Table 1.

	Te *3d_3/2_*:Eu *3d_3/2_*	Te *3d_5/2_*:Eu *3d_3/2_*	Te *3d_3/2_*:Eu *3d_5/2_*	Te *3d_5/2_*:Eu *3d_5/2_*
EuTe_4_:EuTe	3.787	3.933	3.783	3.928

## Data Availability

The data presented in this study are available on request from the corresponding author.

## Supplementary Materials

The following supporting information can be downloaded at: https://www.mdpi.com/article/10.3390/ma16227093/s1, Supplementary Material: A. Optimization of substrate temperature; B. More details on the transmittance spectroscopy.
