# Supplementary material for: Tailoring the Structure and Properties of Epitaxial Europium Tellurides on Si(100) through Substrate Temperature Control
Source: Materials (Basel). 2023 Nov 9;16(22):7093. doi: 10.3390/ma16227093 (PMC10672566; doi:10.3390/ma16227093)
Supplement: Supplementary file 1 [file materials-16-07093-s001.zip › materials-2667567-supplementary.pdf]

materials MDPI

Supplementary Material

Tailoring the Structure and Properties of Epitaxial Europium Tellurides on Si(100) through Substrate Temperature Control

Fan Yu [1], Xiaodong Qiu [1], Jinming Zhou [2], Lin Huang [1], Bin Yang [1], Junming Liu [1,3], Di Wu [1,3], Gan Wang [2,4] and Yi Zhang [1,3,5,*]

[1] National Laboratory of Solid State Microstructure, School of Physics, Nanjing University, Nanjing, 210093, China
[2] Department of Physics, and Shenzhen Institute for Quantum Science and Engineering, Southern University of Science and Technology, Shenzhen 518055, China
[3] Collaborative Innovation Center of Advanced Microstructures, Nanjing University, Nanjing, 210093, China
[4] Guangdong Provincial Key Laboratory of Quantum Science and Engineering, Shenzhen 518055, China
[5] Hefei National Laboratory, Hefei 230088, China
* Correspondence: zhangyi@nju.edu.cn

Citation: To be added by editorial staff during production.

Academic Editor: Firstname Lastname

Received: date
Revised: date
Accepted: date
Published: date

Copyright: © 2023 by the authors. Submitted for possible open access publication under the terms and conditions of the Creative Commons Attribution (CC BY) license (https://creativecommons.org/licenses/by/4.0/).

A. Optimization of substrate temperature

The optimal growth temperature ranges are 200~210 °C for $EuTe_4$ and 210~230 °C for EuTe, respectively. In our manuscript, we set the growth temperatures for the two films as 205 °C and 220 °C, respectively. We utilize the RHEED technique to characterize the film quality at different temperatures, which is the most direct method to monitor the film growth quality in MBE.

Figures S1(a)–S1(f) display the RHEED patterns of europium telluride films grown at different temperatures. From these figures, we can infer:

Figure S1(a), < 190 °C: At this substrate temperature, which is below the condensation temperature of the Te element, a large amount of unreacted Te clusters directly condenses on the substrate, resulting in extremely low film quality.

Figure S1(b), 190–200 °C: The temperature at this point is slightly below the optimum for $EuTe_4$ growth. The RHEED pattern indicates the film is still $EuTe_4$, but its sharpness is not as good as the one grown at 205 °C. This is because the temperature is too low for a sufficient reaction between Eu and Te.

Figure S1(c), 200~210 °C: This is the optimal growth temperature for $EuTe_4$.

Figure S1(d), 210~230 °C: This is the optimal growth temperature for EuTe.

Figure S1(e), 230~250 °C: At this point, sharp RHEED streaks begin to display transmission spots, indicating the presence of some three-dimensional island growth.

Figure S1(f), >250 °C: The RHEED diffraction pattern shows clear transmission spots, indicating that three-dimensional island growth is dominant.

Since this study primarily focuses on the preparation of flat two-dimensional films, we have set the growth temperatures for $EuTe_4$ and EuTe at 205 °C and 220 °C, respectively.

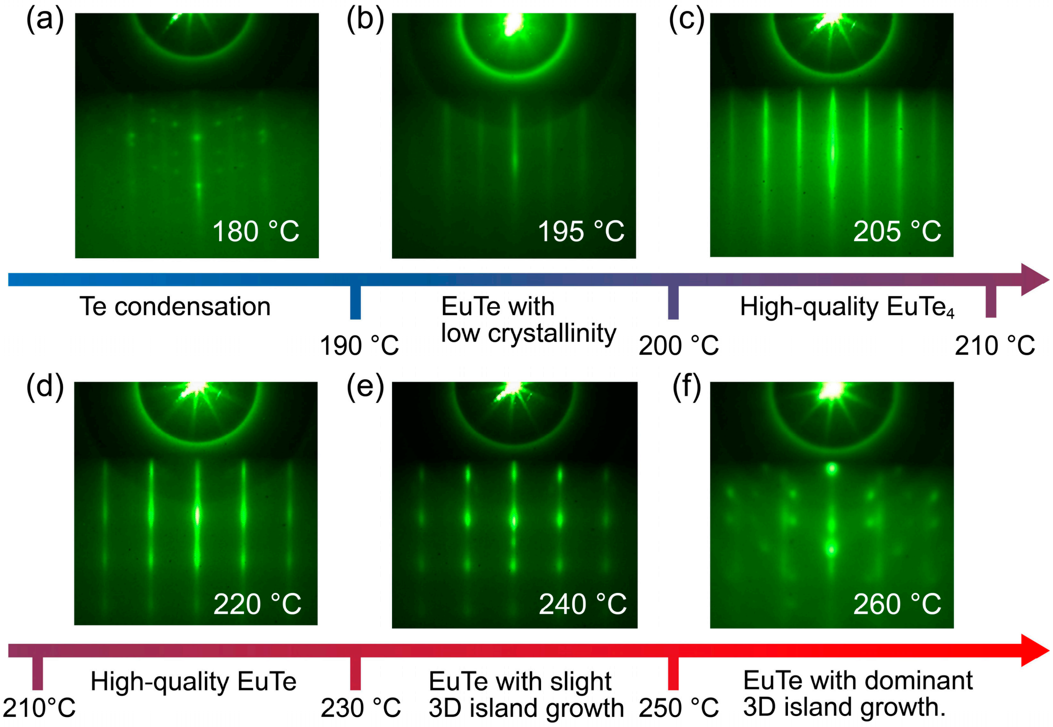

Figure S1. RHEED patterns of EuTe films grown at different substrate temperatures.

B. More details on the transmittance spectroscopy

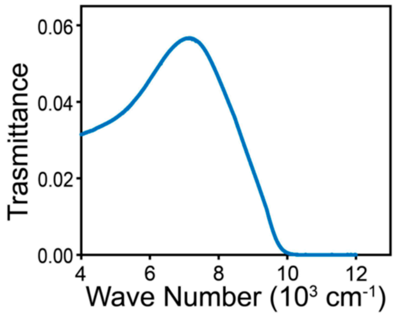

Figure S2. Product of the transmittance spectrum of the silicon substrate and the experimental apparatus (T1=Tsub×Tappa**).**
